# Time-Series Transcriptomic Analysis of Contrasting Rice Materials under Heat Stress Reveals a Faster Response in the Tolerant Cultivar

**DOI:** 10.3390/ijms24119408

**Published:** 2023-05-28

**Authors:** Haiya Cai, Hongpan Wang, Lei Zhou, Bo Li, Shuo Zhang, Yonggang He, Ying Guo, Aiqing You, Chunhai Jiao, Yanhao Xu

**Affiliations:** 1 Hubei Key Laboratory of Food Crop Germplasm and Genetic Improvement, Key Laboratory of Crop Molecular Breeding, Ministry of Agriculture and Rural Affairs, Food Crops Institute, Hubei Academy of Agricultural Sciences, Wuhan 430064, China; caihy@hbaas.com (H.C.); zhoulei@hbaas.com (L.Z.); zhangshuo@hbaas.com (S.Z.); whuhyg@hbaas.com (Y.H.); guokkkyyy@163.com (Y.G.); aq_you@163.com (A.Y.); 2 Scientific Observation and Experiment Station for Crop Gene Resources and Germplasm Enhancement in Hubei, Ministry of Agriculture and Rural Affairs, Wuhan 430064, China; 3 College of Agriculture, Yangtze University, Jingzhou 434025, China; hongpanwang2009@163.com (H.W.); 201973043@yangtzeu.edu.cn (B.L.)

**Keywords:** RNA-seq, dynamic response, MAPK signaling pathway, candidate gene

## Abstract

Short-term heat stress can affect the growth of rice (*Oryza sativa* L.) seedlings, subsequently decreasing yields. Determining the dynamic response of rice seedlings to short-term heat stress is highly important for accelerating research on rice heat tolerance. Here, we observed the seedling characteristics of two contrasting cultivars (T11: heat-tolerant and T15: heat-sensitive) after different durations of 42 °C heat stress. The dynamic transcriptomic changes of the two cultivars were monitored after 0 min, 10 min, 30 min, 1 h, 4 h, and 10 h of stress. The results indicate that several pathways were rapidly responding to heat stress, such as protein processing in the endoplasmic reticulum, glycerophospholipid metabolism, and plant hormone signal transduction. Functional annotation and cluster analysis of differentially expressed genes at different stress times indicate that the tolerant cultivar responded more rapidly and intensively to heat stress compared to the sensitive cultivar. The MAPK signaling pathway was found to be the specific early-response pathway of the tolerant cultivar. Moreover, by combining data from a GWAS and RNA-seq analysis, we identified 27 candidate genes. The reliability of the transcriptome data was verified using RT-qPCR on 10 candidate genes and 20 genes with different expression patterns. This study provides valuable information for short-term thermotolerance response mechanisms active at the rice seedling stage and lays a foundation for breeding thermotolerant varieties via molecular breeding.

## 1. Introduction

Climate change has led to a decline in global food production [1]. It has been predicted that the extreme annual daily maximum temperature will increase by approximately 1 to 4 °C by 2100 [2]. Rice (*Oryza sativa* L.) is the main food crop species worldwide and feeds more than half of the global population [3]. The production of rice will need to increase by 70% in 2050 to satisfy the demand of a rapidly growing global population [4]. According to statistical data, heat stress has caused a 25% reduction in rice yield, and continued warming could pose a serious threat to global production [5]. It has been predicted that each degree-Celsius increase in global mean temperature would, on average, reduce global yields of rice by 3.2% [6].

Plants can reduce the damage caused by heat stress and maintain normal growth via stress signaling [7], ion transport [8], hormone metabolism [9], and transcriptional regulation [10]. As highly conserved serine and threonine protein kinases, mitogen-activated protein kinases (MAPKs) can transmit extracellular stimuli ultimately to elicit intracellular responses [11,12,13]. Plants can induce a primary heat stress response (HSR) via Ca^2+^-dependent calmodulin (CaM3) and H_2_O_2_-induced MAPKs to repair and refold damaged proteins [10,14]. Like WRKY and HSF activities, the MAPK cascade also regulates abscisic acid (ABA) biosynthesis and transcription factor (TF) phosphorylation, which in turn regulate the response to abiotic stress [15,16,17]. Previous studies have reported that the expression of MAPK-related genes plays a key role in the plant heat response. Knocking out SlMAPK3 in tomato plants has been shown to enhance heat tolerance [11]. Similarly, overexpression of maize *ZmMAPK1* in *Arabidopsis* thaliana was shown to increase the survival rate of seedlings under heat stress [18].

Several heat-responsive genes have been identified and characterized in rice in recent years. Three rice QTLs, namely qHTSF4.1, miR169r-5p, and qEMF3, can regulate plant heat tolerance at the flowering stage [19,20,21]. Overexpression of  *HTH5* was shown to increase the seed set at the heading stage of rice plants under heat stress, whereas suppression of *HTH5* resulted in greater susceptibility to heat stress [22]. *TT1*, the thermotolerance gene identified in African rice (*Oryza glaberrima*), encoding an α2 subunit of the 26S proteasome, protects cells from heat stress through the elimination of cytotoxic denatured proteins and maintenance of heat-response processes [23]. *TT2*, encoding a Gγ subunit, confers thermotolerance to rice during both the vegetative and reproductive growth stages without a yield penalty [24]. Experiments involving overexpression and knockout rice lines showed that *HTG3a* could also positively regulate heat tolerance at these stages [25]. Furthermore, the *TT3* natural allele or genetic editing in rice enhanced the heat tolerance and reduced the decrease in grain yield caused by heat stress [26]. Using molecular cloning of *TT1-2* and performing a phenotypic analysis, researchers bred a new hybrid, Zhehangyou 1586, which showed high yield, good quality, and strong heat tolerance [27]. Interestingly, *TT1* [23], *TT2* [24], and *TT3* [26] were identified at the seedling stage and favored yields.

Transcriptomic analysis contributed to revealing the early response mechanism and identified the early stress signal perception genes [28]. Transcriptomic analysis of muskmelons after 15 min salt treatment indicated that calcium-dependent protein kinase (CDPK) was closely related to early abiotic stress response [29]. The overexpression of a rice *OsCPK12* calmodulin-independent protein kinase has been shown to reduce the accumulation of ROS [30]. The early signal perception mechanisms could induce downstream functional genes that are needed to establish new cellular homeostasis in order to lead the plant drought resistance [31]. However, very little is known about the early events in the perception signals of rice seedlings under heat stress.

Monitoring the RNA-seq time-series data can identify the dynamic characteristics of gene regulatory network models and candidate genes [32,33]. The transcriptomic analysis of the *Pyropia chauhanii* monospore-producing process over a series of seven time points revealed that genes related to GTPase activity, signal transduction, and protein phosphorylation may positively regulate the formation and release of monospores [34]. A high-temporal-resolution transcriptomic investigation performed using two foxtail millet (*Setaria italica*) contrast cultivars under drought stress revealed the temporal drought-response process and provided a reference for selecting drought-resistance candidate genes [35]. Furthermore, researchers have identified defense potential candidate genes in response to *Fusarium udum* infection by researching the characteristics of pigeon pea root tissue using this method [36]. The putative seed-dormancy gene AGL was identified based on the gene expression trends according to the juvenile olive-tree time-series transcriptome analysis [37]. Therefore, monitoring the time-series transcriptome would be conducive to better understanding the regulatory mechanism of biological processes.

According to the World Meteorological Organization, many parts of the world have experienced temperatures of 40 °C during the rice seedling stage in recent years (https://worldweather.wmo.int/, accessed on 1 February 2021). This temperature is higher than the optimal temperature for the growth of rice seedlings (25–28 °C), resulting in seedlings being stunted, withering, and even dying [4,38]. Here, we detected the phenotypic changes of two contrasting genotypes of rice, T11 (heat-tolerant) and T15 (heat-sensitive), under different heat stress durations at the seedling stage. The transcriptomic changes of the two cultivars were monitored after 0 min, 10 min, 30 min, 1 h, 4 h, and 10 h of heat stress to better reveal the molecular signatures and patterns of rice seedling short-term heat stress. Furthermore, we also incorporated genome-wide association study (GWAS) analysis results [3] to identify potential candidate genes involved in heat tolerance.

## 2. Results

### 2.1. Phenotype Characteristics of T11 and T15 under Heat Stress

**Figure 1:**
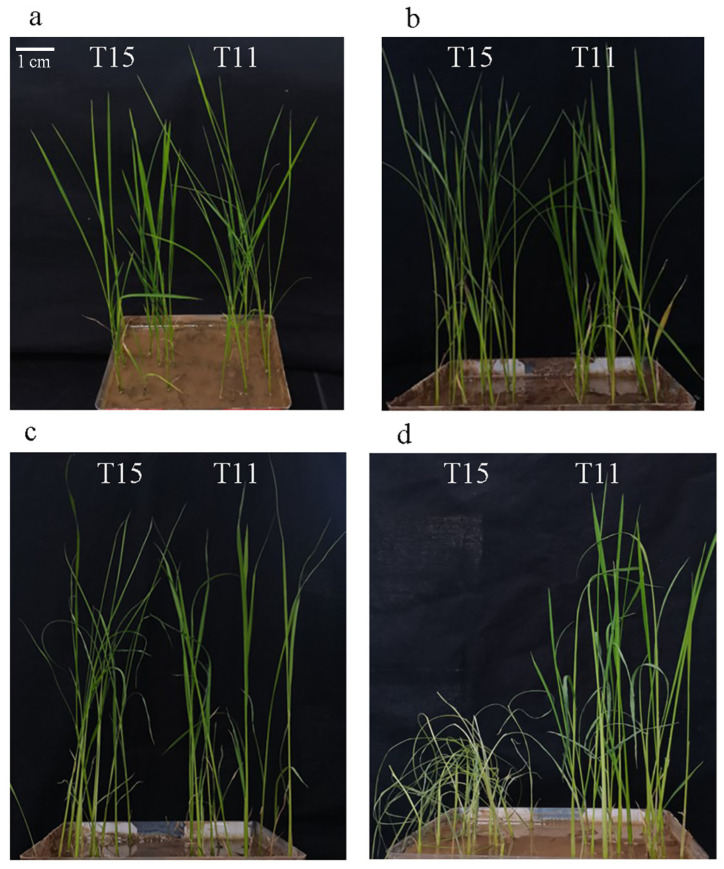
Phenotypic changes of heat-stressed T11 and T15 seedlings. (**a**) T11 and T15 seedlings at 0 h of heat treatment. (**b**) T11 and T15 seedlings at 4 h of heat treatment. (**c**) T11 and T15 seedlings at 10 h of heat treatment. (**d**) T11 and T15 seedlings at 20 h of heat treatment.

Both rice cultivars were green and robust before heat stress (Figure 1a). After applying the heat treatment (42 °C), we found that T15 exhibited some drooping leaves after 4 h stress, and the leaf drooping was even severe for extended heat treatment (Figure 1b). For T15, heat stress-treated seedlings visibly showed a wilting phenotype at 10 h compared to the control seedlings (Figure 1c). Significant differences were evident between T11 and T15 after 20 h of heat treatment, with T15 being nearly dead but T11 having slightly curled leaves (Figure 1d).

Based on these results, T11 showed superior heat tolerance at the seedling stage. To characterize the molecular response mechanisms before and after the phenotypic differences in the rice seedlings, transcriptome analysis was performed on the seedlings at the 0 min, 10 min, 30 min, 1 h, 4 h, and 10 h time points.

### 2.2. RNA–Seq Data Processing and Identification of Differentially Expressed Genes in Response to Heat Stress

**Figure 2:**
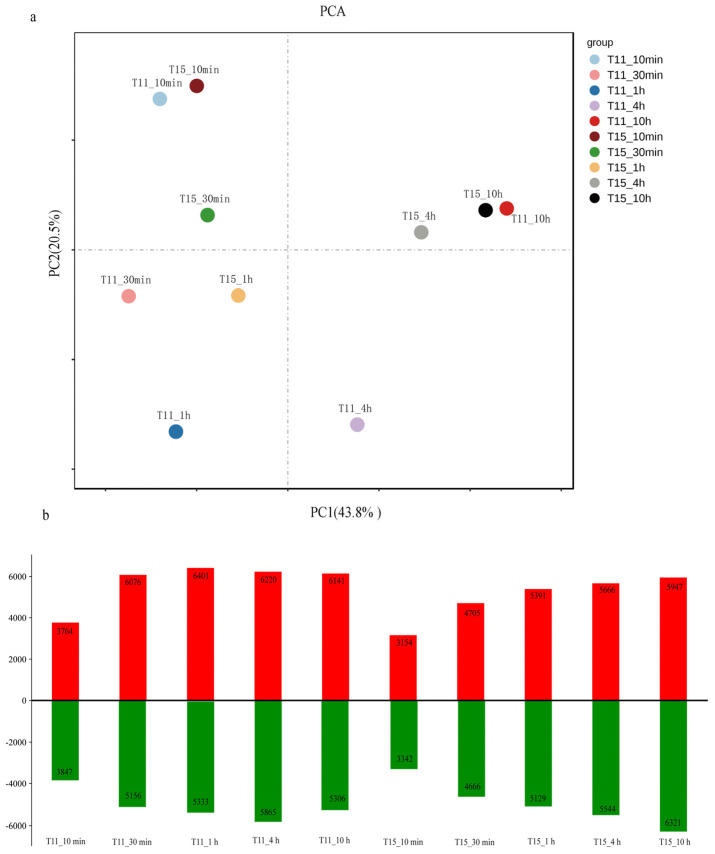
DEGs in the two cultivars in response to heat stress and cluster analysis results. (**a**) PCA of T11 and T15 transcriptome data for samples taken at five different stress times. (**b**) Number of upregulated and downregulated DEGs in T11 and T15 after different heat stress durations.

A total of 243.05 Gb of clean data were obtained from 36 libraries. The clean reads of all samples showed 91.80–95.22% mapping to the reference rice genome. All of the correlation coefficients between the three biological replicates for each treatment based on all of the transcripts were more than 0.9, indicating that the expression data were highly reproducible (Figure S1). We determined the heat-responsive genes by comparing heat-treated samples with the controls for each cultivar at five stress time points (10 min, 30 min, 1 h, 4 h, and 10 h). Principal component analysis (PCA) was conducted to examine time-point-related transcriptional changes in T11 and T15 after heat treatment (Figure 2a). Remarkably, more differentially expressed genes (DEGs) were detected for T11 than for T15 at the first four stress time points, while the opposite was true at 10 h of treatment (Figure 2b, Table S1). The gene expression changed in T11 and T15 after 10 min heat treatment, and we speculate that rice seedlings initially regulate gene depression to resist heat stress. We speculate, based on PCA, that the rice seedlings may have suffered serious damage at the molecular level at 20 h for both cultivars. Moreover, the expression levels of T15 after 4 h of stress and 10 h of stress were relatively similar, which could be related to its sensitivity.

### 2.3. Early-Stage Response of Rice Seedlings under Heat Stress

**Figure 3:**
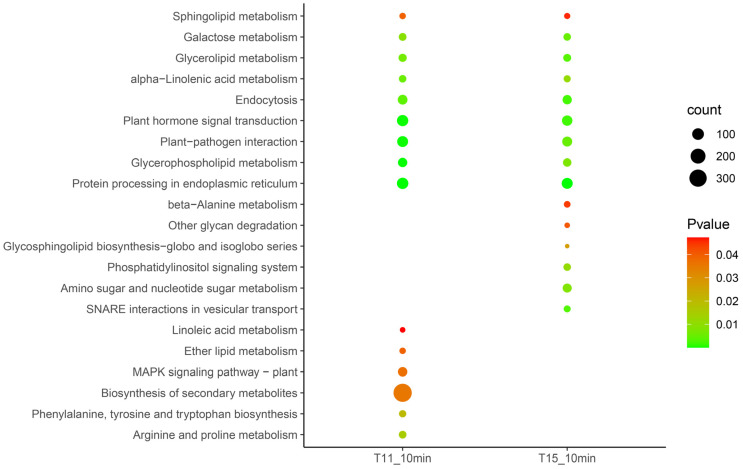
KEGG analysis of differentially expressed genes in response to heat at the early stage.

The Kyoto Encyclopedia of Genes and Genomes (KEGG) annotations were used to resolve the resistance mechanism of rice seedlings at an early stage using the DEGs at 10 min in T11 and T15, respectively (Figure 3). The results showed that protein processing in the endoplasmic reticulum, glycerophospholipid metabolism, plant-pathogen interactions, plant hormone signal transduction, endocytosis, alpha-linolenic acid metabolism, glycerolipid metabolism, galactose metabolism, and sphingolipid metabolism were activated in both T11 and T15. Moreover, we found that the plant MAPK signaling pathway, biosynthesis of secondary metabolites, and arginine and proline metabolism were specifically induced under heat stress in T11. Soluble NSF attachment protein receptor (SNARE) interactions in vesicular transport, amino sugar and nucleotide sugar metabolism, and the phosphatidylinositol signaling system were specifically induced in the T15 cultivar.

### 2.4. Time-Series Transcriptome Expression Trend Analysis

**Figure 4:**
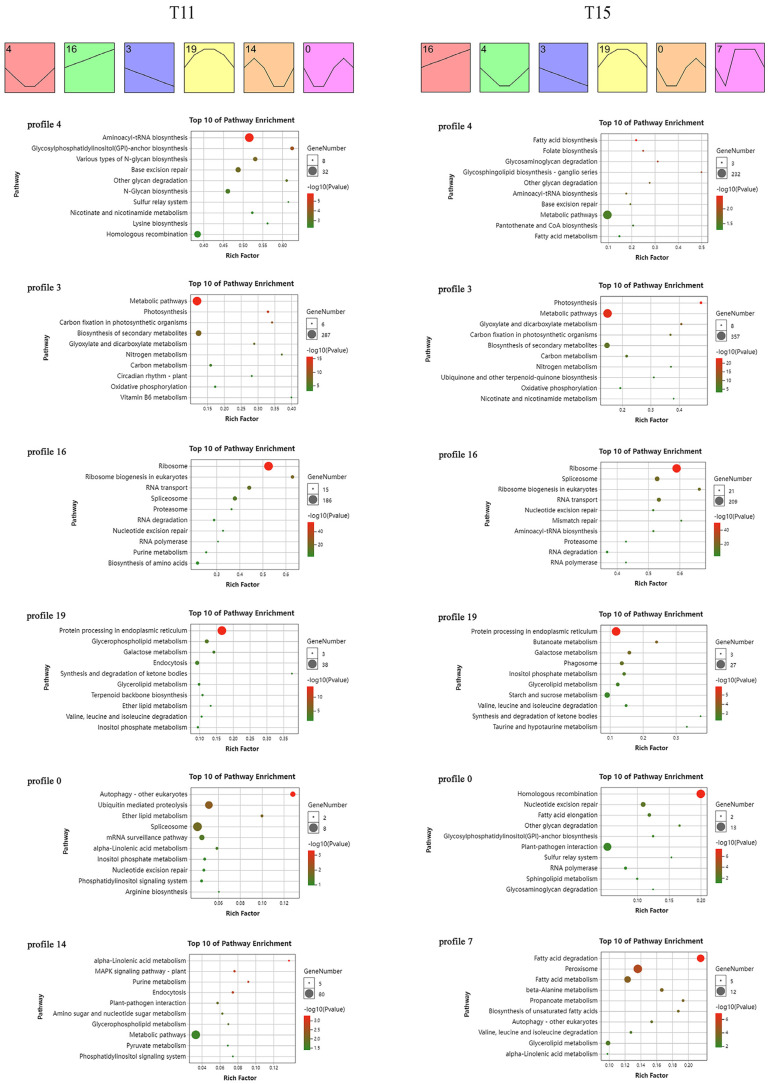
Trend analysis of DEGs at different stress time points and DEG profile functional annotations for T11 and T15.

To gain dynamic insight into heat-induced changes at the gene expression level under heat stress, we performed the time-series transcriptome expression trends analysis using the DEGs in T11 and T15 (Figure 4). There were six significant expression trend profiles in T11 (*p* < 0.05). Profile 4 had the highest significance, followed by profile 16, profile 3, profile 19, profile 14, and profile 0. There were also six significant expression trend profiles in T15: profile 16, profile 4, profile 3, profile 19, profile 0, and profile 7 in descending order of significance. Remarkably, the same profiles (profile 4, profile 16, profile 3, profile 19, and profile 0) had different significance levels and DEG numbers in the two cultivars. We performed KEGG analysis using the DEGs in these profiles to decode the transcriptional regulatory pathways that take place in plants following heat stress.

Profile 4 in T11 (6860 genes) and T15 (2534 genes) included genes that were downregulated rapidly but exhibited a rebound in expression as the stress was prolonged. These genes were enriched in aminoacyl-tRNA biosynthesis, base excision repair, and other glycan degradation activity. Profile 16 (T11 with 3855 genes, T15 with 5377 genes) included genes that were rapidly and continuously upregulated in response to heat stress, and these genes were mainly involved in ribosomes, ribosome biogenesis in eukaryotes, RNA transport, spliceosome, proteasome, RNA degradation, nucleotide excision repair, and RNA polymerase. Profile 3 (T11 with 2228 genes, T15 with 2506 genes) genes were rapidly upregulated and continuously expressed, and they were mainly involved in metabolic pathways, photosynthesis, carbon fixation in photosynthetic organisms, biosynthesis of secondary metabolites, glyoxylate and dicarboxylate metabolism, nitrogen metabolism, carbon metabolism, and oxidative phosphorylation. Profile 19 (T11 with 897 genes, T15 with 1109 genes) genes were upregulated rapidly and then downregulated after reaching a peak in the middle stage. These genes were mainly enriched in protein processing in the endoplasmic reticulum; galactose metabolism; synthesis and degradation of ketone bodies; glycerolipid metabolism; and valine, leucine, and isoleucine degradation. Remarkably, profile 0 genes exhibited the same expression trend but without the same pathways as those in T11 (508 genes) and T15 (992 genes). The genes in this profile were downregulated rapidly, then upregulated, and then downregulated again at the late stage. The genes in T11 were enriched in autophagy, ubiquitin-mediated proteolysis, ether lipid metabolism, the spliceosome, and the mRNA surveillance pathway. The genes in T15 were mainly enriched in homologous recombination, nucleotide excision repair, and fatty acid elongation.

Furthermore, profile 14 (555 genes) genes were responsible for the T11-specific expression trend. These genes were induced rapidly and showed upregulation first but then downregulation, and their expression level rose slightly at the late stage. These genes were enriched in the plant MAPK signaling pathway, purine metabolism, endocytosis, metabolic pathways, and pyruvate metabolism. Among these pathways, the plant MAPK signaling pathway and pyruvate metabolism were specific. Profile 7 (795 genes) genes were responsible for the T15-specific expression trend. These genes were downregulated, upregulated to the highest level, and stably downregulated again. The genes in this profile were mainly enriched in fatty acid degradation and peroxisome and fatty acid metabolism.

### 2.5. Identification of DEGs and Their Functional Annotations between Two Cultivars with Contrasting Heat Tolerances

**Figure 5:**
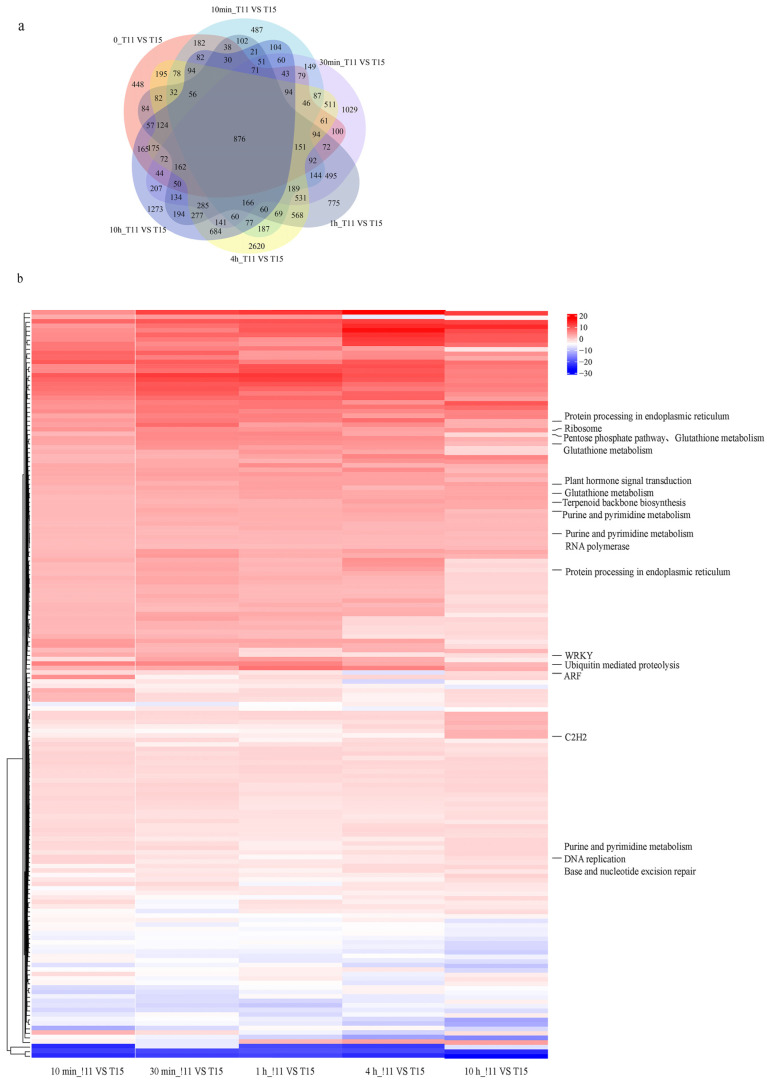
Genes differentially expressed between two cultivars and functional annotations. (**a**) Venn diagrams of DEGs in T11 vs. T15 across five heat treatment time points. (**b**) Interspecific DEG expression levels and functional annotations.

Differences in genetic background were identified by comparing the gene expression between the two cultivars at 0 h. Differences in genetic background were also excluded to examine transcriptional changes in the two cultivars after heat treatment. In total, 2038, 4239, 4061, 6512, and 3794 genes were differentially expressed in T11 and T15 at 10 min, 30 min, 1 h, 4 h, and 10 h under heat stress, respectively. We identified 166 common DEGs between T11 and T15 across all heat treatment time points (Figure 5a), and remarkably, most of these genes were upregulated in T11. Differential expression of these genes may explain the heat-tolerant phenotype of T11, and we predicted TFs and annotated the functions of these genes (Figure 5b). The results showed that three genes were predicted to encode TFs: ARF, C2H2, and WRKY. In addition, the functional annotation results showed that some genes were also involved in plant hormone signal transduction, ubiquitin-mediated proteolysis, protein processing in the endoplasmic reticulum, ribosomes, glutathione metabolism, purine and pyrimidine metabolism, and base and nucleotide excision repair. The expression levels of genes involved in these pathways at the five stress time points revealed that the expression in T11 was higher than that in T15. These factors may have contributed to T11’s heat tolerance.

### 2.6. Plant MAPK Signaling Pathway in Response to Heat Stress

**Figure 6:**
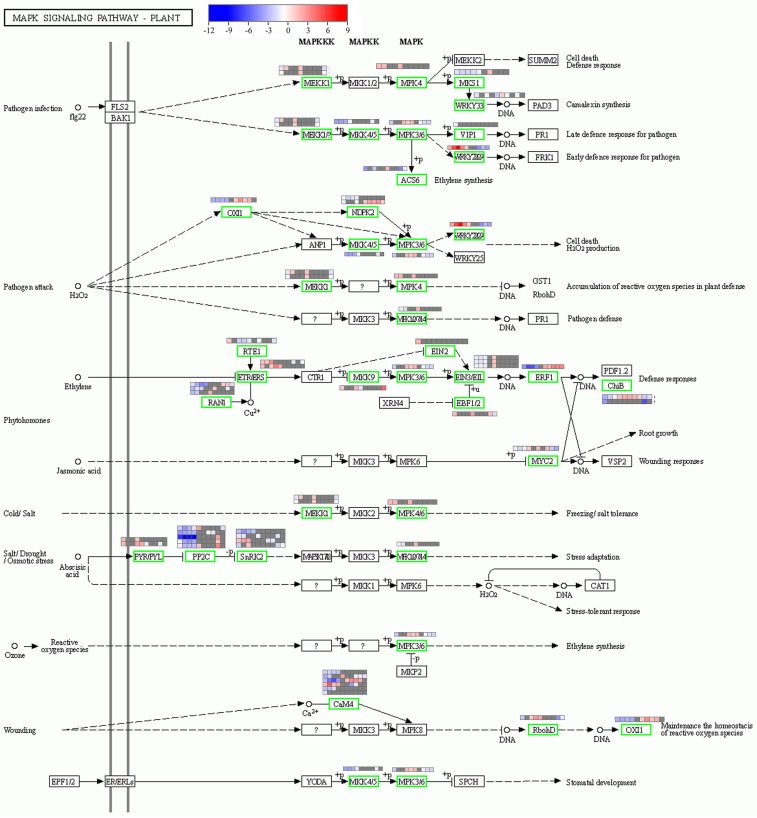
Plant MAPK signaling pathway and the heatmap of the related genes under different stress times points for the heat-tolerant cultivar (T11) and between cultivars (T11 vs. T15). The heat map from left to right means T11_10 min vs. T11_ck, T11_30 min vs. T11_ck, T11_1 h vs. T11_ck, T11_4 h vs. T11_ck, T11_10 h vs. T11_ck, T11_10 min vs. T15_ 10 min, T11_30 min vs. T15_30 min, T11_1 h vs. T15_1 h, T11_4 h vs. T15_4 h, and T11_10 h vs. T15_T11_10 h. The “?” means it has not yet been resolved.

The MAPK signaling pathway was significantly enriched only in the early stress of the heat-tolerant cultivar. The results revealed a total of 48 DEGs involved in this pathway. To explore the role of this pathway in rice heat tolerance, we evaluated their differential expression at different stress time points for T11 and the differential expression between T11 and T15 at different stress time points (Figure 6). The results revealed that six genes encoded CaM; one was upregulated in T11 under 10 min of stress, and five were downregulated. These genes differed between T11 and T15. At the time point of 10 min of T11 stress, two genes encoding the receptor protein PYR/PYL were upregulated, and five genes encoding phosphatase PP2C and three genes encoding MPK were downregulated. A total of four genes were annotated as encoding the kinase SnRK2: one was upregulated, and three were downregulated. The genes encoding WRKY, MPK, and MKK were induced and upregulated in the T11 cultivar at the early stress stage. Remarkably, the gene encoding WRKY was upregulated across all stress time points in T11, and its expression level was higher than that in T15. All of the genes in this pathway showed significant differences in expression levels between the two cultivars across the different stress time points.

### 2.7. Candidate Genes in Response to Heat Stress at the Rice Seedling Stage

Using data from previously reported quantitative trait loci (QTLs) that regulate the response of rice seedlings to heat stress, we identified corresponding genes and compared them with the T11-specific heat-response genes in our data [3]. A total of 27 candidate genes were identified (Table S2). We performed the expression trend analysis with these candidate genes in T11 and T15. The results showed that there were three expression trends both in T11 and T15, namely upregulated constantly, downregulated constantly, and downregulated and then upregulated. The genes with an upregulated trend in T11 and T15 were uniform. However, the other two gene expression trends differed in the two cultivars. There were four and nine genes downregulated constantly in T11 and T15, respectively. In the downregulated and then upregulated trend, we found 13 and 8 genes for T11 and T15 cultivars, respectively. Annotation of these candidate genes resulted in functions related to pyruvate metabolism; mRNA surveillance pathway; biosynthesis of secondary metabolites; and valine, leucine, and isoleucine degradation. Among these, the gene involved in the pyruvate metabolism pathway encoded pyruvate kinase barrel- and α/β domain-containing proteins. Furthermore, several genes encode auxin-responsive proteins, cytochrome P450, stress-induced proteins, and Hsp20/alpha crystallin family members in response to heat stress. Remarkably, LOC_Os05g23140, a member of the Hsp20/alpha crystallin family, was upregulated more than 1000-fold from 10 min to 4 h of stress. However, under 10 h of stress, this gene was slightly downregulated compared with that of the control. Furthermore, seven candidate genes were not functionally annotated. The expression of all the candidate genes may lead to the improved heat tolerance of T11. Nevertheless, their regulatory mechanisms and functions need to be explored and verified.

### 2.8. RNA-Seq Validation by Quantitative Real-Time PCR (RT-qPCR)

To confirm the accuracy and reproducibility of the RNA-seq results, we selected 10 candidate genes and 20 genes with different expression patterns for RT-qPCR. The expression pattern data obtained via RT-qPCR were highly consistent with the RNA-seq data (Figure S2). These results supported the reliability of the RNA-seq data.

## 3. Discussion

Although heat stress has been shown to clearly alter rice seedling phenotypes and gene expression, there are limited data characterizing the short-time-series characteristics of rice seedlings under heat treatment. In recent years, short-term heat wave shock has become severe, which has hindered crop production [39]. This indicates that characterizing the early stage of heat stress in rice is critical for determining future prevention or mitigation strategies. Therefore, we examined changes in phenotypes and transcription in growing seedlings over a 10 h heat stress period (six time points) at 42 °C.

### 3.1. MAPK Signaling Is Transient and Occurs at an Early Stage of Heat Stress

As expected, the rice seedlings were already responding at the molecular level after stress for 10 min. In the present study, both T11 and T15 rapidly regulated gene expression in response to heat stress, but T11 regulated more DEGs than T15. The genes related to heat shock proteins, plant hormone signaling, and sugar and lipid signaling can be rapidly activated in response to heat stress [40]. The same was found in our research: the protein processing and galactose metabolism-related pathways were activated significantly. Protein processing in the endoplasmic reticulum has been proven to play a key role in the response of maize seedlings to heat stress [41].

Interestingly, the MAPK signaling pathway was only enriched in the tolerant cultivar at the 10 min stress time point in this research. MAPK cascades participate in conserved signaling pathways that transmit extracellular stimuli to produce intracellular responses in eukaryotes [42], and they have been shown to respond to a variety of environmental stimuli, including temperature, reactive oxygen species, and drought, by phosphorylating proteins and thus modifying their activity [16,43]. ABA is a key regulator of abiotic stress in plants, and it has been indicated that overexpression of its receptor PYL5 or PYL8 alone could enhance drought resistance in *Arabidopsis* [44,45,46]; the upregulation of PYR/PYL found in this research may have led to the better heat resistance of T11. The binding of the ABA receptor protein PYR/PYL to ABA inhibits PP2C activity and activates SnRK2 [44]. Activated SnRK2 phosphorylates its downstream target genes and leads to the establishment of the abiotic stress response. We hypothesized that the downregulation of PP2C in this research may be conducive to the enhancement of heat resistance in T11. In addition, MPK3/MPK6, key players in stomatal development, are also involved in the abiotic stress response mediated by ABA [47]. It has been reported that *OsMPK3* can contribute to defense signaling by phosphorylating the TF OsbHLH65 [48]. Furthermore, MAPK may act as a master switch to trigger different genes or enzymes involved in tolerance in wheat under heat stress without affecting grain quality [49]. This finding indicated that this pathway may participate in early heat signaling events, and this deduction has been suggested in other studies [50].

### 3.2. Gene Expression Dynamics of Rice in Response to Continuous Heat Stress

Time-series monitoring of the transcriptome could reveal which pathways the plant regulated in response to heat stress and how the expression of the genes in these pathways is regulated [35]. Several recent studies have reported the transcription dynamics of different plant species in response to abiotic stress [51,52]. Therefore, we selected five stress time points to investigate transcriptional dynamic characteristics of rice seedlings from no visible phenotypic changes to leaf wilting based on the phenotypic changes of rice seedlings under heat stress.

We found that the genes related to nucleotide excision repair were upregulated and that their expression increased continually with stress duration. DNA damage is caused when plants encounter environmental stress, and this damage is gradually aggravated with prolonged heat treatment. In response to stress, plants can rapidly initiate repair mechanisms such as nucleic acid excision [53]. The increase in endoplasmic reticulum activity confers increased stress tolerance to plants, which initiates the unfolded protein response [54]. In plants, the UPR signaling pathway involves two ER membrane-associated TFs, bZIP17 and bZIP28, and is also involved in the RNA-splicing factor IRE1 and its target RNA, bZIP60 [55]. Under short-term stress conditions, signaling from IRE1 activates endocytosis [56]. The expression of endocytosis-related genes also changed dynamically in this study. Plant leaves lose water, resulting in hyperosmosis and leading to endocytosis enhanced with increased heat treatment time [57]. Endocytosis is tightly linked to stress signaling pathways, which can help plants to adapt to the ever-changing environment [58]. Similarly, our results on the expression patterns and functional annotation of candidate genes also showed that the genes encoding cytochrome P450, auxin-responsive protein, stress-induced protein Di19, and RNA recognition motifs were continuously upregulated to resist heat stress. Time-series transcriptomic analysis revealed comprehensive transcriptome dynamics of rice seedlings and enhanced our understanding of the molecular mechanisms of rice heat adaptation. Furthermore, the genes of the profile significantly downregulated by heat stress were enriched in carbon fixation in photosynthetic organisms, photosynthesis, carbon metabolism, and their related pathways. These results suggested that the carbon assimilation system is sensitive to elevated temperatures, which agreed with previous work performed in plants [59].

### 3.3. Candidate Genes of Rice Heat Tolerance

Combining GWAS and RNA-seq can provide both the power and the resolution needed to identify candidate genes and has proven to be more successful than either strategy alone. Researchers combining GWAS results with associated RNA-seq analysis data identified 10 candidate genes related to seed germination at low temperatures in maize [60]. Our transcriptome data combined with Wei’s [3] GWAS data could better exclude genes with different genetic backgrounds in screening for more reliable candidates for heat tolerance. All the candidate genes were activated more than 10-fold at multiple time points after heat stress in heat-resistant cultivars. In addition, these genes were differentially expressed at multiple time points between the two cultivars. This may explain why T11 has better heat resistance than T15.

Cytochrome P450, a member of the oxidoreductase class of enzymes [61], is the first line of an organism’s chemical defense [62] and has been shown to play an important role in abiotic stresses such as heat [63], drought, and salt [64]. Previous studies have identified some genes involved in signal transduction and P450 functional categories that have heat-resistance functions in rice seedlings [65]. The candidate gene LOC_Os05g23140 was annotated as an Hsp20/alpha crystallin family member, which may also be involved in the rice heat response. Members of this gene family have been shown to be heat-tolerant in potatoes and wheat [66,67]. The candidate gene LOC_Os05g11140, encoding protein tyrosine kinase, has been predicted to respond to heat stress [68]. Some candidate genes also may play an important role in heat tolerance, although these genes have not been functionally annotated. The function and mechanism of all the candidate genes will be further explored.

## 4. Materials and Methods

### 4.1. Plant Material, Heat Treatment, and Sample Preparation

Two Indica rice varieties, T11 (Erjiunan 1, heat-tolerant) and T15 (Zhegangu, heat-sensitive), were used in our research. Seeds of the two rice cultivars were allowed to germinate on wet filter paper for 4 days and then evenly sown in a small plastic crucible and cultured in an artificial climate chamber (AGC-MR, Zhejiang, China). The culture conditions were as follows: 14 h/28 °C day and 10 h/25 °C night cycle and 250 μmol m^2^s^−1^ light. After 21 days, the seedlings were placed in an incubator at 42 °C. Leaf samples were harvested after 0 min, 10 min, 30 min, 1 h, 4 h, and 10 h of heat treatment, immediately put in liquid nitrogen, and then stored at −80 °C until further analysis. Three biological replicates were included for each time point of each cultivar.

### 4.2. Total RNA Extraction, Library Construction, Sequencing, and Bioinformatic Analysis

Total RNA was extracted from leaves using TRIzol reagent (Invitrogen, Waltham, MA, USA) according to the manufacturer’s instructions. Three biological replicates were collected for each treatment. RNA concentration and purity were measured using a NanoDrop 2000 (Thermo Fisher Scientific, Wilmington, NC, USA). RNA integrity was assessed using an RNA Nano 6000 Assay Kit and an Agilent Bioanalyzer 2100 system (Agilent Technologies, Santa Clara, CA, USA). A total amount of 1 μg of RNA per sample was used as input material for the RNA sample preparations. Sequencing libraries were generated using the NEBNext Ultra RNA Library Prep Kit for Illumina (NEB, Ipswich, MA, USA) following the manufacturer’s recommendations. The library preparations were sequenced, and 150 bp paired-end reads were generated. After filtering using the Fastp Toolkit, all the clean reads were aligned to the reference sequences of MSU v7.0 [69]. Fragments per kilobase million reads (FPKM) were used to quantify the levels of gene expression. Pearson’s correlation coefficient was used to calculate the correlation coefficients of FPKM of all transcripts among three replications for each sample. DESeq2 was used for differential expression analysis based on count value, and clustered profiles of genes with a *p* value ≤ 0.05 and |log_2_(fold-change)| ≥ 1 were considered differentially expressed [70]. STEM was conducted to classify the identified DEG expression patterns. KEGG pathway analysis of the DEGs was performed using the OmicShare tools, a free online platform for data analysis (http://www.omicshare.com/tools, accessed on 7 February 2023). The gene Nr function was annotated based on the NCBI nonredundant protein sequence database. The online platform PlantTFDB v5.0 (http://planttfdb.gao-lab.org/, accessed on 7 February 2023) was used for TF analysis. Venn diagrams and heatmaps were constructed using TBtools software V1.098 [71].

### 4.3. RT–qPCR for RNA-Seq Validation

The RNA samples used for RT-qPCR were identical to those used for the RNA-seq experiments to validate the reliability. Primers (Table S3) were designed using Primer v5 software (Premier Biosoft International, San Francisco, CA, USA), and ubiquitin (UBQ5) was used as an internal control. The experimental operating system used for RT-qPCR was described in our previous studies [70,72]. The data were processed according to the Ct (2^−ΔΔCt^) method.

## 5. Conclusions

We explored the mechanisms of heat response and tolerance at different biological levels of seedlings of the two different rice cultivars upon high-temperature exposure. Our data suggested that although the phenotype did not change significantly in the early stage of stress, its gene expression levels changed in response to heat stress. Compared with the heat-sensitive one, the heat-tolerant cultivar can rapidly regulate more genes to cope with heat stress. This research investigated the transcriptome characteristics of two rice cultivars at six different stress time points at the seedling stage, the results of which reveal the dynamic molecular response mechanism. We also identified 27 candidate genes for rice heat tolerance. This enhances our understanding of the molecular mechanisms of plant heat adaptation and provides a resource for heat tolerance candidate genes. In future work, we will concentrate on the functional verification of these candidate genes and explore how they function in response to heat stress.

## Data Availability

The raw transcriptome sequencing data generated and used for analysis in this study are deposited in the NCBI Sequence Read Archive (SRA) database (Bioproject PRJNA822001) as per the NCBI submission guidelines.

## Supplementary Materials

The following supporting information can be downloaded at: https://www.mdpi.com/article/10.3390/ijms24119408/s1.
