# Supplementary figures and images for: Time-Series Transcriptomic Analysis of Contrasting Rice Materials under Heat Stress Reveals a Faster Response in the Tolerant Cultivar
Source: Int J Mol Sci. 2023 May 28;24(11):9408. doi: 10.3390/ijms24119408 (PMC10253628; doi:10.3390/ijms24119408)

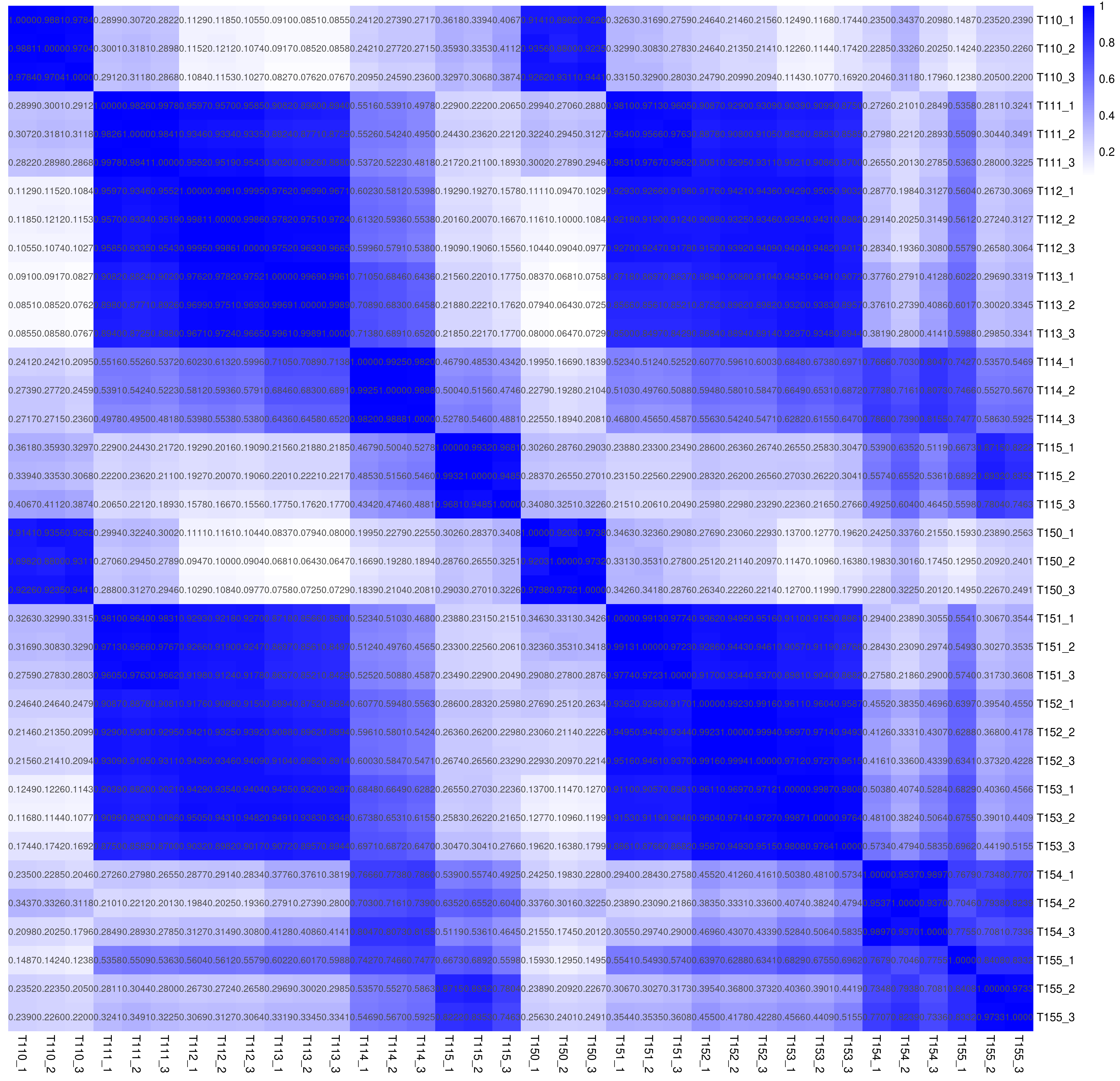

Supplement: Supplementary file 1 [file ijms-24-09408-s001.zip › Supplementary/Figure S1.png]

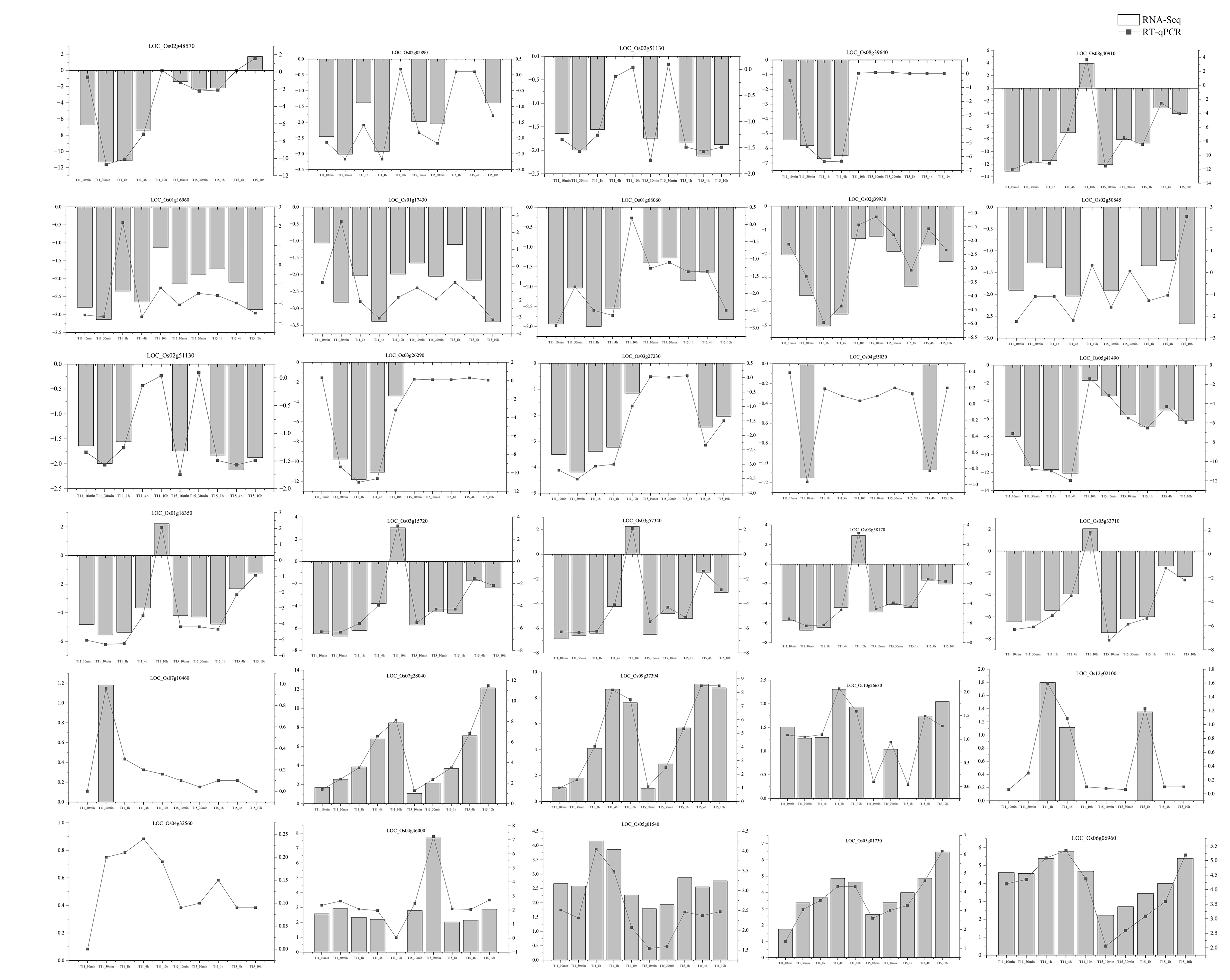

Supplement: Supplementary file 1 [file ijms-24-09408-s001.zip › Supplementary/Figure S2.png]
